# Increased risk of maternal and neonatal complications in hormone replacement therapy cycles in frozen embryo transfer

**DOI:** 10.1186/s12958-020-00601-3

**Published:** 2020-05-04

**Authors:** Liping Zong, Peihao Liu, Liguang Zhou, Daimin Wei, Lingling Ding, Yingying Qin

**Affiliations:** 1 Center for Reproductive Medicine, Shandong University, No.157 Jingliu Road, Jinan, China; 2 National Research Center for Assisted Reproductive Technology and Reproductive Genetics, Jinan, China; 3 The Key Laboratory of Reproductive Endocrinology (Shandong University) Ministry of Education, Jinan, China; 4 Shandong Provincial Hospital Affiliated to Shandong University, Jinan, China

**Keywords:** Frozen embryo transfer, Endometrial preparation, Maternal and neonatal complications, Natural cycle, Hormone replacement therapy, Ovulation induction cycle

## Abstract

**Background:**

The endometrial preparation during frozen embryo transfer (FET) can be performed by natural cycle (NC), hormone replacement therapy (HRT) cycle and cycle with ovulation induction (OI). Whether different FET preparation protocols can affect maternal and neonatal outcomes is still inconclusive.

**Methods:**

This was a retrospective cohort study that included 6886 women who delivered singleton live birth babies after 28 weeks of pregnancy underwent FET from January, 2015 to July, 2018. Women were divided into three groups according to the protocols used for endometrial preparation during FET: NC group (*N* = 4727), HRT group (*N* = 1642) and OI group (*N* = 517).

**Results:**

After adjusting for the effect of age, body mass index (BMI), irregular menstruation, antral follicle count (AFC), endometrial thickness, the levels of testosterone, anti-Müllerian hormone (AMH), preconceptional fasting glucose (PFG), systolic and diastolic pressure et al., the HRT group had higher risk of hypertensive disorders of pregnancy (HDP) compared with the NC group (adjusted odds ratio (aOR) 2.00, 95% confidence interval (CI) 1.54–2.60). Singletons born after HRT FET were at increased risk of low birth weight (LBW) compared to NC group (aOR 1.49, 95%CI 1.09–2.06). The risks of preterm birth (PTB) in the HRT and OI group were elevated compared with the NC group (aOR 1.78, 95%CI 1.39–2.28 and aOR 1.51, 95%CI 1.02–2.23, respectively).

**Conclusions:**

The HRT protocol for endometrial preparation during frozen embryo transfer of blastocysts was associated with increased risk of maternal and neonatal complications, compared to the NC and OI protocol.

## Introduction

In 1984, the success of first live birth after thawing the frozen human embryos was reported by the team of Zeilmaker [1]. Since then, the proportion of frozen embryo transfer (FET) has been increasing in in-vitro fertilization (IVF) along with the development of laboratory techniques for cryopreservation and the reduced number of embryo transfer in fresh cycle [2]. Recently, the so called “freeze-all strategy”, i.e. selectively freezing all embryos and performing FET later has become an optimal choice in cycles with high risk of ovarian hyperstimulation syndrome (OHSS), preimplantation genetic testing (PGT) and double ovarian stimulation (DuoStim) protocols [3]. Several studies reported that FET achieved higher pregnancy rates and lower complications rates compared with fresh embryo transfer [4–6]. Therefore, the “freeze-all strategy” has been widely used to improve live birth rates and decrease potential complications.

Multiple protocols for endometrium preparation have been explored during FET. The common protocols include natural cycles (NC), artificial cycle with hormone replacement therapy (HRT), and cycle with ovulation induction (OI) [7, 8]. Groenewoud et al. conducted a randomized controlled trial (RCT) concluding that HRT cycle was not inferior to modified natural cycle for FET with regard to live birth rates (LBRs), clinical and ongoing pregnancy rates [9]. The latest Cochrane review suggested there were no significant differences in pregnancy rates, miscarriage rates, or live birth rates among different endometrial preparation protocols for FET [7]. However, the effect of different protocols on maternal and neonatal outcomes is still uncertain. Here, we performed a retrospective study to compare the maternal and neonatal outcomes in women underwent NC, HRT, or OI protocol for endometrial preparation.

## Materials and methods

### Study design and participants

**Fig. 1:**
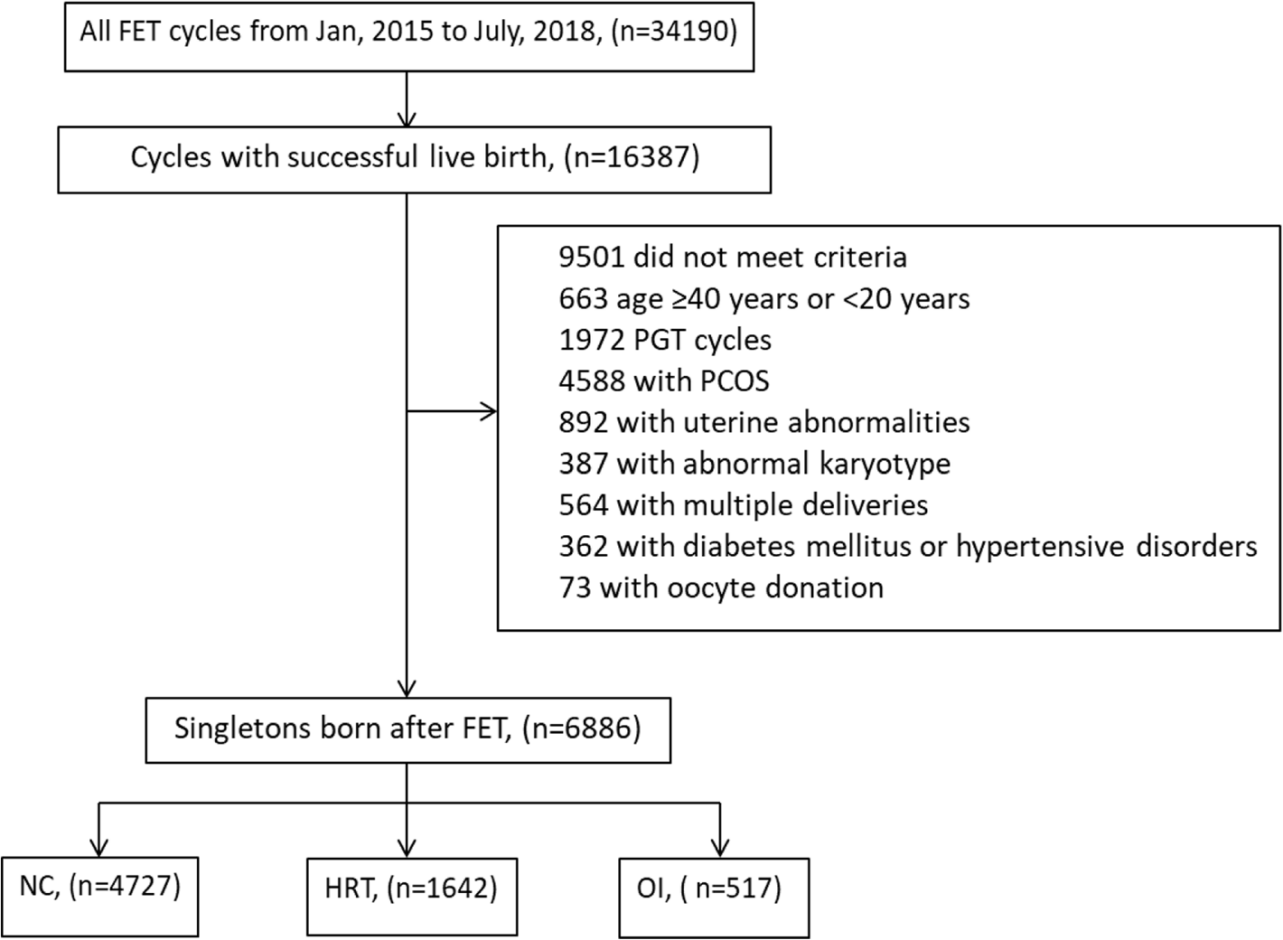
Workflow of Study Enrollment

The study included 6886 women aged 20–40, who received FET treatment after IVF/ intracytoplasmic sperm injection (ICSI) cycles in Center for Reproductive Medicine, Shandong University from January, 2015 to July, 2018 and delivered singleton live birth baby after 28 weeks of pregnancy. These women were divided into three groups according to the endometrial preparation protocols: NC group (*n* = 4727), HRT group (*n* = 1642) and OI group (*n* = 517). Women with type II diabetes mellitus or preconceptional fasting glucose (PFG) ≥7.0 mmol/L, preconceptional hypertension, polycystic ovary syndrome (PCOS), uterine malformation and intrauterine adhesion were excluded. PCOS was defined as menstrual abnormalities (irregular uterine bleeding, oligomenorrhea, or amenorrhea) combined with either hyperandrogenism or polycystic ovaries [10]. PGT and oocyte donation cycles were also excluded. All blastocysts were vitrified on day 5 or day 6 according to the embryo development. (Fig. 1, Additional file 1: Table S1).

### Endometrial preparation protocols for FET

In general, NC protocol was the preferred choice for women with regular menstruation. For patients with irregular menstruation or history of oligo-ovulation or anovulation, HRT or OI protocol was used as first choice.

In the NC group, transvaginal ultrasound was starting since day 10–12 of the menstrual cycle depending on the diameter of the follicle till ovulation. Urinary luteinizing hormone (LH) was tested combined with the ultrasound examination when the dominant follicle reached 14 mm in diameter. Human chorionic gonadotrophin (hCG, Le Baode, Livzon) was administrated to imitate the LH surge when the diameter of dominant follicle reaching 18 mm or more. Embryo transfer was scheduled 5 days after ovulation. Thirty mg oral dydrogesterone (Duphaston, Abbott Biologicals B.V.) was administered daily from ovulation until the 12th week of pregnancy.

In the HRT group, patients were prescribed with 4 mg oral estradiol valerate (Progynova, Delpharm Lille) since day 2–4 of menstruation for 5–6 days, and then 6 mg for the following 5–6 days. Endometrium thickness was monitored after 10–12 days of medication by transvaginal ultrasound along with the serum levels of LH, estradiol (E_2_) and progesterone (P). Thereafter the dose of estradiol valerate, which was 8 mg/d maximally, was modulated according to the endometrium thickness and the E_2_ levels. When the endometrium thickness reached at least 7 mm, FET was scheduled in 5 days. Dydrogesterone 40 mg/d and progesterone capsules (Utrogestan, Capsugel) 200 mg/d was given as luteal phase support until the 12th week of pregnancy. Meanwhile, 6 mg/d to 8 mg/d of estradiol valerate was continued until clinical pregnancy, which was defined as the presence of an intrauterine gestational sac by ultrasonography at 7–8 weeks of gestation.

In the OI group, 75 IU/d of human menopausal gonadotropin (HMG, Le Baode, Livzon) was started on day 3–5. Dose of HMG was adjusted according to the development of follicles as monitored on ultrasonography and the measurement of serum sex steroids. Urinary hCG was administrated at dose of 6000 IU to 8000 IU when one or two follicles reached 18 mm or more in diameter.

One or two blastocysts were transferred. Maternal and neonatal complications including hypertensive disorders of pregnancy (HDP), gestational diabetes mellitus (GDM), placenta previa, oligohydramnios, preterm birth (PTB), low birth weight (LBW), small/large for gestational age (SGA/LGA) and gender of neonates were analyzed. HDP included gestational hypertension, pre-eclampsia, eclampsia and HELLP syndrome. GDM was diagnosed according to the 2013 WHO criteria (fasting plasma glucose ≥5.1 mmol/l; and/or 1 h plasma glucose ≥10.0 mmol/l; and/or 2 h plasma glucose ≥8.5 mmol/l after 24 weeks of gestation) [11]. Placenta praevia referred to a placenta that lay in close proximity to the internal cervical os or may partially or completely cover it after 28 weeks of gestation. Oligohydramnios was defined as the single deepest pocket (SDP) ≤ 2 cm or amniotic fluid index (AFI) ≤ 5 cm. PTB was defined as delivery before 37 gestational weeks while not earlier than 28 gestational weeks [12]. LBW referred to birth weight of full-term delivered newborns below 2500 g. SGA was defined as birth weight below the 10th percentile referential birth weight; LGA was defined as birth weight higher than the 90th percentile referential birth weight [13].

### Statistical analysis

Continuous variables were summarized as the mean ± standard deviation and were compared by One-way ANOVA test. Chi-square test was used to compare the maternal and neonatal outcomes among the three groups. Multivariate logistic regression was performed to adjust for the effect of age, body mass index (BMI), irregular menstruation, the use of donor sperm, FET cycle number, number of transferred embryos, vanishing twin gestation, preconceptional fasting glucose (PFG), systolic pressure, diastolic pressure, endometrial thickness, antral follicle count (AFC), testosterone level, anti-Müllerian hormone (AMH) on HDP, PTB and LBW. Statistical significance level was set at 0.05. All statistical analyses were performed with SPSS (SPSS Inc., Version 21.0, Chicago, USA).

## Results

The number of women receiving NC, HRT and OI protocol for FET were 4727, 1642 and 517, respectively. The baseline characteristics were listed in Table 1. BMI (23.2 ± 3.4 vs. 22.8 ± 3.3 vs. 22.5 ± 3.2 kg/m^2^, *p* < 0.001), AFC (15.6 ± 6.6 vs. 15.3 ± 6.0 vs. 14.9 ± 5.9, *p* < 0.001), and levels of testosterone (26.0 ± 11.9 vs. 25.6 ± 12.5 vs. 24.4 ± 11.7 ng/dL, *p* < 0.001) and AMH (5.3 ± 3.6 vs. 5.0 ± 3.5 vs. 4.7 ± 3.7 ng/mL, *p* < 0.001) were higher in HRT group compared to OI and NC group. As expected, HRT group and OI group had higher rates of irregular menstruation compared with NC group (21.9% vs. 21.7% vs. 5.6%, *p* < 0.001). Systolic pressure (120.8 ± 11.6 vs. 120.7 ± 11.9 vs. 119.5 ± 11.8, *p* < 0.001) and diastolic pressure (72.4 ± 8.6 vs. 73.3 ± 8.8 vs. 71.7 ± 8.7, *p* < 0.001) were higher in HRT and OI groups than NC group. And difference was also found in the FET cycle number among NC, HRT and OI groups (1.2 ± 0.5, 1.3 ± 0.6, 1.6 ± 0.8, *p* < 0.001). However, no difference was observed in the rate of donor sperm using (9.3% vs. 8.0% vs. 7.4%, *p* = 0.115) and vanishing twin gestation (3.0% vs. 3.2% vs. 2.5%, *p* = 0.710) among the three groups.

**Table 1:** The baseline characteristics of all participants

	NC *N* = 4727	HRT *N* = 1642	OI *N* = 517	*p*-value
Maternal age (year)	30.8 ± 4.0	30.5 ± 4.1	30.9 ± 4.1	0.109
BMI (kg/m^2^)	22.5 ± 3.2^a,b^	23.2 ± 3.4	22.8 ± 3.3	< 0.001^*^
Indications for IVF				
Tubal factor	2960 (62.6)	1025 (62.4)	325 (62.9)	0.981
Male factor	1056 (22.3)	356 (21.7)	114 (22.1)	0.856
Combined factors	566 (12.0)	201 (12.2)	60 (11.6)	0.919
Others	145 (3.1)	60 (3.7)	18 (3.5)	0.486
Irregular menstruation, n (%)	267 (5.6)^a,b^	359 (21.9)	112 (21.7)	< 0.001^*^
Donor sperm using, n (%)	441 (9.3)	131 (8.0)	38 (7.4)	0.115
FET cycle number	1.2 ± 0.5^a,b^	1.3 ± 0.6^c^	1.6 ± 0.8	< 0.001^*^
No.of transferred embryos, n (%)				
= 1	4138 (87.5)	1455 (88.6)	453 (87.6)	0.516
≥ 2	589 (12.5)	187 (11.4)	64 (12.4)	0.516
Vanishing twin gestation^d^, n (%)	143 (3.0)	53 (3.2)	13 (2.5)	0.710
PFG (mmol/L)	5.2 ± 0.5^b^	5.2 ± 0.5^c^	5.3 ± 0.5	0.002^*^
Systolic pressure (mmHg)	119.5 ± 11.8^a,b^	120.8 ± 11.6	120.7 ± 11.9	< 0.001^*^
Diastolic pressure (mmHg)	71.7 ± 8.7	72.4 ± 8.6	73.3 ± 8.8	< 0.001^*^
Endometrial thickness (mm)	10.0 ± 1.6^a,b^	9.5 ± 1.5^c^	9.3 ± 1.7	< 0.001^*^
AFC	14.9 ± 5.9^a^	15.6 ± 6.6	15.3 ± 6.0	< 0.001^*^
Testosterone level (ng/dL)	24.4 ± 11.7^a,b^	26.0 ± 11.9	25.6 ± 12.5	< 0.001^*^
AMH^e^ (ng/mL)	4.7 ± 3.7^a^	5.3 ± 3.6^c^	5.0 ± 3.5	< 0.001^*^

*NC* natural cycle, *HRT* hormone replacement therapy, *OI* ovulation induction, *BMI* body mass index, *FET* frozen embryo transfer, *PFG* preconceptional fasting glucose, *AFC* Antral follicle count, *AMH* anti-müllerian hormone

*There were significant differences among the three groups

^a^There were significant differences between NC and HRT group

^b^There were significant differences between NC and OI group

^c^There were significant differences between HRT and OI group

^d^5 of the patients underwent selective reduction of triplet or quadruplet pregnancy. 7 of the patients were of triplet pregnancy yet lost two fetuses spontaneously during early pregnancy

^e^There were data missing in AMH, including 430 in NC group and 122 in HRT group and 39 in OI group

Table 2 listed the maternal and neonatal outcomes. For the maternal outcomes, women using HRT protocol had higher rates of HDP (7.9% in HRT vs. 4.6% in OI vs. 3.5% in NC, *p* < 0.001) than women in OI and NC group. Significant difference was observed in the rates of GDM among the three groups (6.5% in HRT vs. 7.5% in OI vs. 5.2% in NC, *p* = 0.030). The subgroup analyses showed that the GDM rate in OI group was higher than that in NC group, however, no difference existed between HRT group and NC group. The rates of placenta previa (1.5% in HRT vs. 1.2% in OI vs. 1.0% in NC, respectively, *p* = 0.299) and oligohydramnios (1.0% in HRT vs. 1.7% in OI vs. 1.3% in NC, respectively, *p* = 0.437) were similar among the three groups. For the neonatal outcomes (Table 2), HRT and OI groups had higher risk of PTB compared to NC group (7.9% in HRT vs. 4.6% in NC, *p* < 0.001 and 7.7% in OI vs. 4.6% in NC, *p* = 0.001), but no significant difference was found between HRT and OI groups (7.9% vs. 7.7%, *p* = 0.773). The HRT group had increased risk of LBW compared with the NC group (4.5% vs. 2.8%, *p* = 0.001); however, no significant difference was observed between the OI group and the NC group (3.7% vs. 2.8%, *p* = 0.289). In addition, different LGA risk was found among three groups (26.2% in HRT vs. 22.1% in OI vs. 23.4% in NC, *p* = 0.040), although there was no significant difference between HRT and NC groups (*p* = 0.112). Additionally, the three groups were comparable in terms of SGA risk (2.9% in HRT vs. 5.0% in OI vs. 3.5% in NC, *p* = 0.073) and gender of neonates (*p* = 0.890).

**Table 2:** Maternal and neonatal outcomes after FET in women with NC, HRT and OI group

	NC	HRT	OI	*P*-value
**Maternal outcomes**				
HDP, n. (%)	166 (3.5)^a^	130 (7.9)^c^	24 (4.6)	< 0.001^*^
GDM, n. (%)	247 (5.2)^b^	106 (6.5)	39 (7.5)	0.030^*^
Placenta previa, n. (%)	47 (1.0)	24 (1.5)	6 (1.2)	0.299
Oligohydramnios, n. (%)	61 (1.3)	17 (1.0)	9 (1.7)	0.437
**Neonatal outcomes**				
Gestational age (weeks)				
< 32	39 (0.6)^b^	18 (1.1)	8 (1.5)	0.023^*^
32–36^+ 6^	188 (4.0)^a,b^	111 (6.8)	32 (6.2)	< 0.001^*^
≥ 37	4510 (95.4)^a,b^	1513 (92.1)	477 (92.3)	< 0.001^*^
LBW, n. (%)	130 (2.8)^a^	74 (4.5)	19 (3.7)	0.002^*^
SGA, n. (%)	165 (3.5)	48 (2.9)	26 (5.0)	0.073
LGA, n. (%)	1104 (23.4)	430 (26.2)^c^	114 (22.1)	0.040^*^
Gender of neonates n. (%)				0.890
Male, n. (%)	2500 (52.9)	863 (52.6)	278 (53.8)	
Female, n. (%)	2227 (47.1)	779 (47.4)	239 (46.2)	

*FET* frozen embryo transfer, *NC* natural cycle, *HRT* hormone replacement therapy, *OI* ovulation induction, *HDP* hypertensive disorders of pregnancy, *GDM* gestational diabetes mellitus, *LBW* low birth weight, *SGA* small for gestational age, *LGA* large for gestational age

*There were significant differences among the three groups

^a^ There were significant differences between NC and HRT group

^b^ There were significant differences between NC and OI group

^c^ There were significant differences between HRT and OI group

**Fig. 2:**
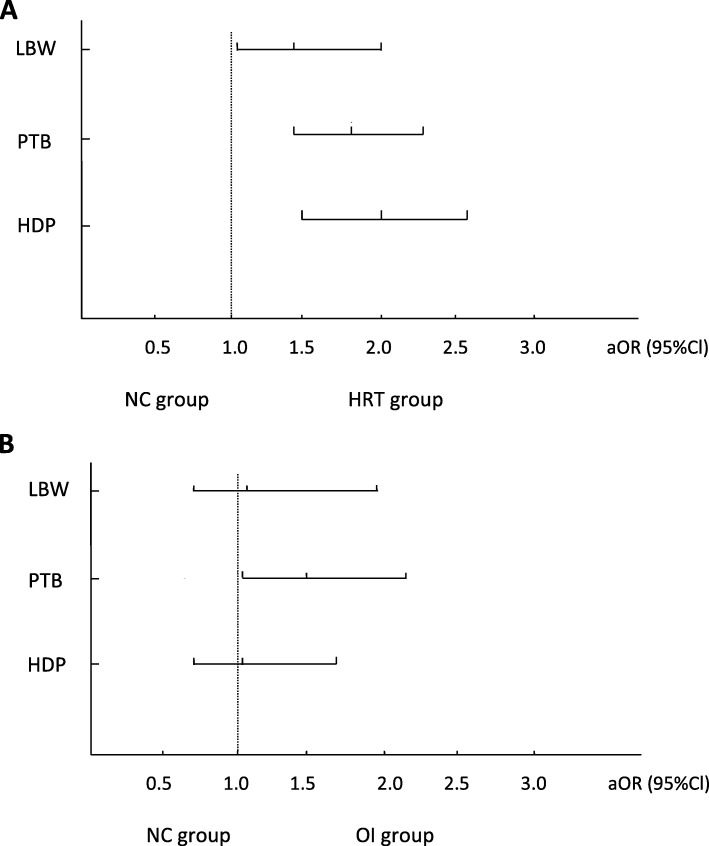
Comparison of HDP, PTB and LBW between groups. Adjusted odds ratio (aOR) of hypertensive disorders of pregnancy (HDP), preterm birth (PTB), and low birth weight (LBW) compared women with natural cycles (NC) group. **a** aOR in women with hormone replacement therapy (HRT) group compared with NC group. **b** aOR in women with ovulation induction (OI) group compared with NC group

After adjusting for the effect of age, BMI, irregular menstruation, AFC, endometrial thickness, the levels of testosterone, AMH, PFG, systolic and diastolic pressure et al. (Table 3, Fig. 2), HRT group still showed higher risk of HDP (aOR 2.00, 95% CI 1.54–2.60) and LBW (aOR 1.49, 95%CI 1.09–2.06) compared with the NC group. Risk of PTB in the HRT and OI groups were elevated compared with NC group (aOR 1.78, 95%CI 1.39–2.28 and aOR 1.51, 95%CI 1.02–2.23, respectively).

**Table 3:** Univariate and multivariate logistic regression model about NC, HRT and OI protocols

	Crude OR (95% Cl)	*P*-value	Adjusted OR (95% Cl)	*P*-value
**HDP**				
NC	1		1	
HRT	2.36 (1.86–2.99)	< 0.001^*^	2.00 (1.54–2.60)	< 0.001^*^
OI	1.34 (0.86–2.07)	0.193	1.02 (0.62–1.65)	0.853
**GDM**				
NC	1		1	
HRT	1.25 (0.99–1.58)	0.061	1.10 (0.84–1.45)	0.470
OI	1.48 (1.04–2.10)	0.028^*^	1.49 (0.97–2.19)	0.056
**Placenta previa**				
NC	1		1	
HRT	1.48 (0.90–2.42)	0.122	1.52 (0.88–2.64)	0.133
OI	1.17 (0.50–2.75)	0.720	1.35 (0.55–3.27)	0.514
**Oligohydramnios**				
NC	1		1	
HRT	0.80 (0.47–1.37)	0.419	0.70 (0.40–1.24)	0.225
OI	1.36 (0.67–2.75)	0.399	1.06 (0.49–2.29)	0.887
**PTB**				
NC	1		1	
HRT	1.84 (1.47–2.30)	< 0.001^*^	1.78 (1.39–2.28)	< 0.001^*^
OI	1.76 (1.24–2.50)	0.002^*^	1.51 (1.02–2.23)	0.041^*^
**LBW**				
NC	1		1	
HRT	1.67 (1.25–2.23)	0.001^*^	1.49 (1.09–2.06)	0.014^*^
OI	1.35 (0.83–2.20)	0.231	1.17 (0.70–1.96)	0.547
**SGA**				
NC	1		1	
HRT	0.83 (0.60–1.15)	0.271	0.85 (0.60–1.21)	0.370
OI	1.46 (0.96–2.24)	0.078	1.47 (0.94–2.31)	0.092
**LGA**				
NC	1		1	
HRT	1.16 (1.02–1.33)	0.021^*^	1.15 (0.99–1.33)	0.058
OI	0.93 (0.75–1.16)	0.505	0.98 (0.77–1.24)	0.842

*NC* natural cycle, *HRT* hormone replacement therapy, *OI* ovulation induction, *OR* odds ratio, *CI* confidence interval, *HDP* hypertensive disorders of pregnancy, *GDM* gestational diabetes mellitus, *PTB* preterm birth, *LBW* low birth weight, *SGA* small for gestational age, *LGA* large for gestational age

Adjustment included age, body mass index, irregular menstruation, donor sperm using, FET cycle number, number of transferred embryos, vanishing twin gestation, preconceptional fasting glucose, systolic pressure, diastolic pressure, endometrial thickness, antral follicle count, testosterone level, anti-Müllerian hormone

*There were significant differences between groups

The comparisons between IVF and ICSI cycles were listed in Additional file 2: Table S2-S13.

## Discussion

In this study, we compared the maternal and neonatal outcomes of singletons born after FET with different endometrial preparation protocols in a large cohort. A higher risk of HDP and LBW was observed in the HRT group, as well as increased risks of PTB in both HRT and OI group compared with NC group. The differences in the maternal and neonatal outcomes could be explained by the excessive estrogen exposal, absence of corpus luteum (CL) and distinct clinical characteristics of population in the HRT group.

During early pregnancy, the uterine spiral arteries transform from high-resistance, low-capacity to low-resistance, high-capacity vessels by trophoblasts immigrating, invading and replacing the endothelial and smooth muscle wall of the spiral arteries [14, 15]. The remodeling of the uterine spiral arteries is crucial for sufficient nutrient and oxygen supply from the placenta to the fetus through an optimal uteroplacental blood flow. Excessive estrogen levels have been reported to impair the invasion of trophoblastic vessels during pregnancy. In pregnant baboon, it has been shown that estrogen plays a major role in regulating morphological and functional differentiation of the villous trophoblasts and signals between the placenta and fetus. Moreover, an elevated estrogen level markedly suppressed the vascular invasion [16–18]. Attenuation of trophoblast vascular invasion and spiral artery restructure will result in placental defect, which subsequently interferes the pregnancy process and end up with complications such as hypertensive and growth disorders [19–23].

It has been evidenced that high levels of estrogen could increase the risks of maternal and neonatal outcome during IVF-ET treatment. Imudia et al. discovered that an elevated serum E_2_ concentration during controlled ovarian hyperstimulation (COH) was associated with higher risk of maternal preeclampsia and SGA newborns for fresh embryo transfer [24]. Pereira N et al. conducted a retrospective cohort study of 4071 patients undergoing fresh IVF-ET cycles, indicating that serum E_2_ levels exceeding 2500 pg/ml during COH seemed to be an independent predictor for LBW in full-term singletons [25]. Additionally, compared to FET, fresh embryo transferred showed worse obstetrical outcome, including LBW and PTB, which was also attributed to a hyperestrogenic milieu generated during ovarian stimulation [26–29].

During the preparation with HRT protocol, the patients were prescribed estrogen normally from 2 weeks before embryo transfer till 8 ^th^ -10th week of pregnancy. It is assumed that the intake of exogenous estrogens during the period of trophoblastic vessels invasion may result in increased risk of maternal and neonatal complications, such as HDP and LBW. Study from Tatsumi et al. compared the pregnancy and neonatal outcomes among OI with letrozole cycle, natural cycle and HRT cycle for FET and discovered differences in terms of gestational weeks at delivery, birth weight and SGA/LGA among three groups [30].

Another explanation for high risk of HDP in HRT protocol could be ascribed to the absence of corpus luteum (CL) in the first trimester when CL contributed most to hormone secretion. According to a recent study by von Versen-Höynck F, CL defect was associated with elevated rate of preeclampsia, which was probably caused by lack of circulating relaxin, a potent vasodilator secreted by CL yet not supplemented in the luteal phase support [31]. Another study also stressed that the vascular health was impaired when no CL was present during early pregnancy, which suggested an insufficient vascular adaption involved in the development of preeclampsia [32]. Ginström Ernstad E et al. conducted a population-based retrospective study in Sweden, in which FET cycles were grouped according to presence or absence of a CL [33]. His results demonstrated an increased risk of hypertensive disorders in HRT FET cycles, which was in accordance with our findings.

Thirdly, the increased risk of obstetric and neonatal complications in the HRT group was also caused by the distinct characteristics of the population. In our study, the preferred choice for endometrium preparation was NC protocol; while for patients with irregular menstruation or history of oligo-ovulation or anovulation, HRT or OI will be suggested. Although patients with PCOS had been excluded in the study, it was till reasonable to expect more women with endocrine disturbance in HRT and OI group than NC group. Not unexpectedly, compared to the NC group, women in HRT group had more AFC, higher BMI, and serum testosterone levels. Increased BMI and high levels of testosterone were involved in obstetrical complications through altered trophoblast invasion and placentation [34]. Obese patients were more likely to have dyslipidemia, which was associated with pregnancy complications and adverse pregnancy outcomes owing to vascular damage and endothelial dysfunction caused by oxidative stress from free radicals and lipid peroxides [34–36]. Hyperandrogenism and insulin resistance were also reported to alter endovascular trophoblast invasion and placentation [37–39]. Excess maternal androgens reduced placental weight and affected fetal growth in rats [40]. Therefore, the endocrine-metabolic disturbance in women using HRT protocol may also contributed to the adverse pregnancy outcomes.

Our sample size was large enough to detect the differences of maternal and neonatal complications in different groups as well as to adjust for the maternal characteristics. One of the limitations was the estradiol concentration during early pregnancy was unavailable thus the comparisons of estradiol level during pregnancy among different FET groups were lacking. Another problem was that since OI protocol was costlier and more time intensive for patient visiting compared to HRT protocol, patients in the OI group might tend to have a history of thin endometrium or cancelled cycles which could lead to different maternal characteristics in this group.

## Conclusion

Hormone replacement therapy protocol for frozen embryo transfer of blastocysts may be associated with adverse maternal and neonatal outcomes, such as high risks of HDP, LBW and PTB. Our results provide reference for endometrial preparation during FET treatment. More attention should be paid to the potential harmful effects of excessive estrogen and corpus luteum defect on maternal and neonatal complications during pregnancy.

## Supplementary information

**Additional file 1: Table S1.** Indications for frozen embryo transfer

**Additional file 2: Table S2-S13.** The comparisons between IVF and ICSI cycles in frozen embryo transfer.

## Data Availability

The data sets used and/or analyzed during the current study are available from the corresponding author on reasonable request.

## Abbreviations

FET: Frozen embryo transfer
NC: Natural cycle
HRT: Hormone replacement therapy
OI: Ovulation induction
LBRs: Live birth rates
PFG: Preconceptional fasting glucose
PCOS: Polycystic ovary syndrome
HDP: Hypertensive disorders of pregnancy
GDM: Gestational diabetes mellitus
PTB: Preterm birth
LBW: Low birth weight
SGA: Small for gestational age
LGA: Large for gestational age
BMI: Body mass index
PFG: Preconceptional fasting glucose
AFC: Antral follicle count
AMH: Anti-Müllerian hormone
CL: Corpus luteum
COH: Controlled ovarian hyperstimulation
