# Supplementary material for: Increased risk of maternal and neonatal complications in hormone replacement therapy cycles in frozen embryo transfer
Source: Reprod Biol Endocrinol. 2020 May 4;18:36. doi: 10.1186/s12958-020-00601-3 (PMC7199365; doi:10.1186/s12958-020-00601-3)
Supplement: Supplementary file 1 — Additional file 1: Table S1. Indications for frozen embryo transfer [file 12958_2020_601_MOESM1_ESM.docx]

Supplementary table 1 Indications for frozen embryo transfer

|  | NC (n=2492) | HRT (n=922) | OI (n=267) | *p*-value |
| --- | --- | --- | --- | --- |
| Indications for FET, n (%) |  |  |  |  |
| High OHSS risk | 1812 (72.7)^a^ | 725 (78.6)^c^ | 186 (69.7) | 0.001^*^ |
| Elevated P level | 65 (2.6)^b^ | 24 (2.6)^c^ | 17 (6.4) | 0.002^*^ |
| Hydrosalpinx | 65 (2.6) | 20 (2.2) | 6 (2.2) | 0.741 |
| Patient's personal reasons | 550 (22.1)^a^ | 153 (16.6) | 58 (21.7) | 0.002^*^ |

*Note:* FET= frozen embryo transfer; OHSS = ovarian hyperstimulation syndrome; P= progesterone

*There were significant differences among groups.

^a^ There were significant differences between NC and HRT group.

^b^ There were significant differences between NC and OI group.

^c^ There were significant differences between HRT and OI group.
