# Supplementary material for: Increased risk of maternal and neonatal complications in hormone replacement therapy cycles in frozen embryo transfer
Source: Reprod Biol Endocrinol. 2020 May 4;18:36. doi: 10.1186/s12958-020-00601-3 (PMC7199365; doi:10.1186/s12958-020-00601-3)
Supplement: Supplementary file 2 — Additional file 2: Table S2-S13. The comparisons between IVF and ICSI cycles in frozen embryo transfer. [file 12958_2020_601_MOESM2_ESM.docx]

Supplementary table 2 The baseline characteristics in the NC group

|  | IVF (n=3074) | ICSI (n=1653) | *p*-value |
| --- | --- | --- | --- |
| Maternal age (year) | 30.9±4.0 | 30.5±4.1 | <0.001^*^ |
| BMI (kg/m^2^) | 22.5±3.2 | 22.5±3.3 | 0.721 |
| Indications for IVF, n (%) |  |  |  |
| Tubal factor | 2508 (81.6) | 452 (27.3) | <0.001^*^ |
| Male factor | 163 (5.3) | 893 (54.0) | <0.001^*^ |
| Combined factors | 343 (11.2) | 223 (13.5) | 0.018^*^ |
| Others | 60 (2.0) | 85 (5.1) | <0.001^*^ |
| Irregular menstruation, n (%) | 175 (5.7) | 92 (5.6) | 0.857 |
| Donor sperm using, n (%) | 417 (13.6) | 24 (1.5) | <0.001^*^ |
| FET cycle number | 1.3±0.5 | 1.2±0.5 | 0.026^*^ |
| No.of transferred embryos, n (%) |  |  | 0.007^*^ |
| =1 | 2662 (86.6) | 1476 (89.3) |  |
| ≥2 | 412 (13.4) | 177 (10.7) |  |
| Vanishing twin gestation, n (%) | 104 (3.4) | 39 (2.4) | 0.050 |
| PFG (mmol/L) | 5.2±0.5 | 5.2±0.5 | 0.800 |
| Systolic pressure (mmHg) | 119.0±11.7 | 120.5±11.8 | <0.001^*^ |
| Diastolic pressure (mmHg) | 71.4±8.7 | 72.2±8.6 | 0.004^*^ |
| Endometrial thickness (mm) | 10.0±1.6 | 10.0±1.6 | 0.884 |
| AFC | 14.7±5.9 | 15.3±5.8 | 0.002^*^ |
| Testosterone level (ng/dL) | 24.4±11.8 | 24.4±11.6 | 0.942 |
| AMH (ng/mL) | 4.7±3.4 | 4.7±4.3 | 0.730 |

*Note:* NC = natural cycle; BMI = body mass index; FET = frozen embryo transfer; PFG = preconceptional fasting glucose; AFC = Antral follicle count; AMH = anti-müllerian hormone.

*There were significant differences among groups.

Supplementary table 3 Univariate and multivariate logistic regression between IVF and ICSI in the NC group

|  | IVF | ICSI | Crude OR (95% Cl) | *p*-value | Adjusted OR (95% Cl) | *p*-value |
| --- | --- | --- | --- | --- | --- | --- |
|  | N=3074 | N=1653 |  |  |  |  |
| HDP, n (%) | 114 (3.7) | 52 (3.1) | 0.84 (0.60-1.18) | 0.317 | 0.77 (0.55-1.08) | 0.130 |
| GDM, n (%) | 159 (5.2) | 88 (5.3) | 1.03 (0.79-1.35) | 0.824 | 1.06 (0.80-1.40) | 0.684 |
| Placenta previa, n (%) | 37 (1.2) | 10 (0.6) | 0.50 (0.25-1.01) | 0.052 | 0.55 (0.27-1.11) | 0.096 |
| Oligohydramnios, n (%) | 3.5 (1.1) | 26 (1.6) | 1.39 (0.83-2.31) | 0.209 | 1.24 (0.73-2.09) | 0.428 |
| PTB, n (%) | 149 (4.8) | 66 (4.0) | 0.82 (0.61-1.10) | 0.179 | 0.83 (0.62-1.13) | 0.237 |
| LBW, n (%) | 90 (2.9) | 40(2.4) | 0.82 (0.56-1.20) | 0.309 | 0.82 (0.56-1.20) | 0.307 |
| SGA, n (%) | 99 (3.2) | 66 (4.0) | 1.25 (0.91-1.72) | 0.169 | 1.20 (0.87-1.67) | 0.263 |
| LGA, n (%) | 743 (24.2) | 361 (21.8) | 0.88 (0.76-1.01) | 0.071 | 0.89 (0.76-1.03) | 0.885 |

*Note:* CI = confidence interval; HDP = hypertensive disorders of pregnancy; GDM = gestational diabetes mellitus; PTB = preterm birth; LBW = low birth weight; SGA = small for gestational age; LGA = large for gestational age.

Adjustment included age, indications for IVF, Donor sperm using, FET cycle number, number of transferred embryos, systolic pressure, diastolic pressure, antral follicle count,

Supplementary table 4 The baseline characteristics in the HRT group

|  | IVF (n=1058) | ICSI (n=584) | *p-*value |
| --- | --- | --- | --- |
| Maternal age (year) | 30.8±4.1 | 30.1±4.1 | 0.001^*^ |
| BMI (kg/m^2^) | 23.2±3.3 | 23.1±3.6 | 0.879 |
| Indications for IVF, n (%) |  |  |  |
| Tubal factor | 862(81.5) | 163 (27.9) | <0.001^*^ |
| Male factor | 47 (4.4) | 309 (52.9) | <0.001^*^ |
| Combined factors | 125(11.8) | 76(13.0) | 0.478 |
| Others | 24 (2.3) | 36 (6.2) | <0.001^*^ |
| Irregular menstruation, n (%) | 234 (22.1) | 125 (21.4) | 0.738 |
| Donor sperm using, n (%) | 121 (11.4) | 10 (1.7) | <0.001^*^ |
| FET cycle number | 1.4±0.7 | 1.3±0.6 | 0.007^*^ |
| No.of transferred embryos, n (%) |  |  | 0.088 |
| =1 | 927 (87.6) | 528 (90.4) |  |
| ≥2 | 131 (12.4) | 56 (9.6) |  |
| Vanishing twin gestation, n (%) | 33 (3.1) | 20 (3.4) | 0.737 |
| PFG (mmol/L) | 5.2±0.5 | 5.2±0.4 | 0.310 |
| Systolic pressure (mmHg) | 120.9±11.7 | 120.6±11.4 | 0.679 |
| Diastolic pressure (mmHg) | 72.3±8.8 | 72.6±8.4 | 0.569 |
| Endometrial thickness (mm) | 9.5±1.5 | 9.6±1.5 | 0.305 |
| AFC | 15.2±6.5 | 16.3±6.7 | 0.001^*^ |
| Testosterone level (ng/dL) | 25.9±11.8 | 26.1±11.9 | 0.662 |
| AMH (ng/mL) | 5.3±3.6 | 5.3±3.5 | 0.824 |

*Note:* HRT = hormone replacement therapy; BMI = body mass index; FET = frozen embryo transfer; PFG = preconceptional fasting glucose; AFC = Antral follicle count; AMH = anti-müllerian hormone.

*There were significant differences among groups.

Supplementary table 5 Univariate and multivariate logistic regression between IVF and ICSI in the HRT group

|  | IVF | ICSI | Crude OR (95% Cl) | *P*-value | Adjusted OR (95% Cl) | *P*-value |
| --- | --- | --- | --- | --- | --- | --- |
|  | N=1058 | N=584 |  |  |  |  |
| HDP, n (%) | 90 (8.5) | 40 (6.8) | 0.79 (0.54-1.17) | 0.791 | 0.78 (0.53-1.16) | 0.220 |
| GDM, n (%) | 78 (7.4) | 28 (4.8) | 0.63 (0.41-0.99) | 0.043* | 0.72 (0.45-1.13) | 0.152 |
| Placenta previa, n (%) | 16 (1.5) | 8 (1.4) | 0.91 (0.39-2.13) | 0.818 | 0.97 (0.40-2.32) | 0.968 |
| Oligohydramnios, n (%) | 11 (1.0) | 6 (1.0) | 0.99 (0.36-2.69) | 0.981 | 0.93 (0.33-2.57) | 0.882 |
| PTB, n (%) | 88 (8.3) | 44 (7.5) | 0.90 (0.62-1.31) | 0.576 | 0.92 (0.63-1.36) | 0.675 |
| LBW, n (%) | 45 (4.3) | 29 (5.0) | 1.18 (0.73-1.90) | 0.506 | 1.24 (0.76-2.02) | 0.401 |
| SGA, n (%) | 27 (2.6) | 21 (3.6) | 1.42 (0.80-2.54) | 0.232 | 1.50 (0.83-2.74) | 0.182 |
| LGA, n (%) | 283 (26.7) | 147 (25.2) | 0.92 (0.73-1.16) | 0.487 | 0.92 (0.73-1.17) | 0.490 |

*Note:* CI = confidence interval; HDP = hypertensive disorders of pregnancy; GDM = gestational diabetes mellitus; PTB = preterm birth; LBW = low birth weight; SGA = small for gestational age; LGA = large for gestational age.

*There were significant differences among groups.

Adjustment included age, tubal factor, male factor, others factor, donor sperm using, FET cycle number, antral follicle count.

Supplementary table 6 The baseline characteristics in the OI group

|  | IVF (n=355) | ICSI (n=162) | *p-*value |
| --- | --- | --- | --- |
| Maternal age (year) | 31.0±3.9 | 30.6±4.5 | 0.283 |
| BMI (kg/m^2^) | 22.8±3.3 | 22.8±3.3 | 0.985 |
| Indications for IVF, n (%) |  |  |  |
| Tubal factor | 281 (79.2) | 44 (27.2) | <0.001^*^ |
| Male factor | 31 (8.7) | 83 (51.2) | <0.001^*^ |
| Combined factors | 34 (9.6) | 26 (16.0) | 0.033^*^ |
| Others | 9 (2.5) | 9 (5.6) | 0.082 |
| Irregular menstruation, n (%) | 71 (20.0) | 41 (25.3) | 0.174 |
| Donor sperm using, n (%) | 31 (8.7) | 7 (4.3) | 0.075 |
| FET cycle number | 1.6±0.8 | 1.5±0.7 | 0.267 |
| No.of transferred embryos, n (%) |  |  | 0.379 |
| =1 | 308(86.8) | 145 (89.5) |  |
| ≥2 | 47 (13.2) | 17 (10.5) |  |
| Vanishing twin gestation, n (%) | 9 (2.5) | 4 (2.5) | 0.964 |
| PFG (mmol/L) | 5.3±0.5 | 5.2±0.5 | 0.211 |
| Systolic pressure (mmHg) | 120.3±12.1 | 121.5±11.3 | 0.262 |
| Diastolic pressure (mmHg) | 72.9±8.9 | 74.3±8.4 | 0.093 |
| Endometrial thickness (mm) | 9.2±1.7 | 9.4±1.7 | 0.245 |
| AFC | 15.0±5.8 | 15.9±6.3 | 0.117 |
| Testosterone level (ng/dL) | 25.7±12.1 | 25.3±13.3 | 0.769 |
| AMH (ng/mL) | 5.0±3.4 | 5.0±3.7 | 0.927 |

*Note:* OI = ovulation induction; BMI = body mass index; FET = frozen embryo transfer; PFG = preconceptional fasting glucose; AFC = Antral follicle count; AMH = anti-müllerian hormone.

*There were significant differences among groups.

Supplementary table 7 Univariate and multivariate logistic regression

between IVF and ICSI in the OI group

|  | IVF | ICSI | Crude OR (95% Cl) | *P*-value | Adjusted OR (95% Cl) | *P*-value |
| --- | --- | --- | --- | --- | --- | --- |
|  | N=355 | N=162 |  |  |  |  |
| HDP, n (%) | 18(5.1) | 6 (3.7) | 0.72 (0.28-1.85) | 0.495 | 0.42 (0.13-1.34) | 0.143 |
| GDM, n (%) | 29 (8.2) | 10 (6.2) | 0.74 (0.35-1.56) | 0.427 | 0.63 (0.26-1.55) | 0.314 |
| Placenta previa, n (%) | 5 (1.4) | 1 (0.6) | 0.44 (0.05-3.75) | 0.449 | 0.30 (0.03-3.71) | 0.349 |
| Oligohydramnios, n (%) | 8 (2.3) | 1 (0.6) | 0.27 (0.03-2.17) | 0.218 | 0.21 (0.02-2.28) | 0.202 |
| PTB, n (%) | 26 (7.3) | 14 (8.6) | 1.20 (0.61-2.36) | 0.603 | 1.33 (0.60-2.94) | 0.483 |
| LBW, n (%) | 9 (2.5) | 10 (6.2) | 2.53 (1.01-6.35) | 0.048^*^ | 2.67 (0.92-7.73) | 0.071 |
| SGA, n (%) | 16 (4.5) | 10 (6.2) | 1.39 (0.62-3.14) | 0.423 | 1.58 (0.60-4.17) | 0.353 |
| LGA, n (%) | 76 (21.4) | 38 (23.5) | 1.13 (0.72-1.75) | 0.602 | 1.05 (0.62-1.80) | 0.851 |

*Note:* CI = confidence interval; HDP = hypertensive disorders of pregnancy; GDM = gestational diabetes mellitus; PTB = preterm birth; LBW = low birth weight; SGA = small for gestational age; LGA = large for gestational age.

*There were significant differences among groups.

Adjustment included tubal factor, male factor and combined factors.

Supplementary table 8 The baseline characteristics in the IVF group

|  | NC | HRT | OI | *p*-value |
| --- | --- | --- | --- | --- |
|  | N=3074 | N=1058 | N=355 |  |
| Maternal age (year) | 30.9±4.0 | 30.8±4.1 | 31.0±3.9 | 0.550 |
| BMI (kg/m^2^) | 22.5±3.2^a^ | 23.2±3.3 | 22.8±3.3 | <0.001^*^ |
| Indications for IVF, n (%) |  |  |  |  |
| Tubal factor | 2349 (76.4) | 832 (78.6) | 281 (79.2) | 0.214 |
| Male factor | 163(5.3) | 47 (4.4) | 18(5.1) | 0.547 |
| Combined factors | 502 (16.3) | 155 (14.7) | 47 (13.2) | 0.180 |
| Others | 60 (2.0) | 24 (2.3) | 9 (2.5) | 0.672 |
| Irregular menstruation, n (%) | 175 (5.7) | 234 (22.1) | 71 (20.0) | <0.001^*^ |
| Donor sperm using, n (%) | 417 (13.6)^a^ | 121 (11.4)^c^ | 31 (8.7) | 0.013^*^ |
| FET cycle number | 1.3±0.5^a,b^ | 1.4±0.7^c^ | 1.6±0.80 | <0.001^*^ |
| No.of transferred embryos, n (%) |  |  |  |  |
| =1 | 2662 (86.6) | 927 (87.6) | 308(86.8) | 0.697 |
| ≥2 | 412 (13.4) | 131 (12.4) | 47 (13.2) | 0.697 |
| Vanishing twin gestation, n (%) | 104 (3.4) | 33 (3.1) | 9 (2.5) | 0.668 |
| PFG (mmol/L) | 5.2±0.5^b^ | 5.2±0.5 | 5.3±0.5 | 0.001^*^ |
| Systolic pressure (mmHg) | 119.0±11.7^a,b^ | 120.9±11.7 | 120.3±12.1 | <0.001^*^ |
| Diastolic pressure (mmHg) | 71.4±8.7^a,b^ | 72.3±8.8 | 72.9±8.9 | 0.001^*^ |
| Endometrial thickness (mm) | 10.0±1.6^a,b^ | 9.5±1.5^c^ | 9.3±1.7 | <0.001^*^ |
| AFC | 14.7±5.9^a^ | 15.2±6.5 | 15.0±5.8 | 0.071 |
| Testosterone level (ng/dL) | 24.4±11.8^a^ | 25.9±11.8 | 25.7±12.1 | 0.001^*^ |
| AMH (ng/mL) | 4.7±3.4^a^ | 5.3±3.6 | 5.0±3.4 | <0.001^*^ |

*Note:* NC = natural cycle; BMI = body mass index; FET = frozen embryo transfer; PFG = preconceptional fasting glucose; AFC = Antral follicle count; AMH = anti-müllerian hormone.

*There were significant differences among groups.

^a^ There were significant differences between NC and HRT group.

^b^ There were significant differences between NC and OI group.

^c^ There were significant differences between HRT and OI group.

Supplementary table 9 The comparison of outcomes in the IVF group

|  | NC | HRT | OI | *p*-value |
| --- | --- | --- | --- | --- |
|  | N=3074 | N=1058 | N=355 |  |
| HDP, n (%) | 114 (3.7)^a^ | 90 (8.5)^c^ | 18 (5.1) | <0.001^*^ |
| GDM, n (%) | 159 (5.2)^a,b^ | 78 (7.4) | 29 (8.2) | 0.006^*^ |
| Placenta previa, n (%) | 37 (1.2) | 16 (1.5) | 5 (1.4) | 0.730 |
| Oligohydramnios, n (%) | 35 (1.1) | 11 (1.0) | 8 (2.3) | 0.162 |
| PTB, n (%) | 149 (4.8)^a,b^ | 88 (8.3) | 26 (7.3) | <0.001^*^ |
| LBW, n (%) | 90 (2.9) | 45 (4.3) | 9 (2.5) | 0.081 |
| SGA, n (%) | 79 (3.2) | 27 (2.6) | 16 (4.5) | 0.181 |
| LGA, n (%) | 743 (24.2) | 283 (26.7) | 76 (21.4) | 0.087 |

*Note:* HDP = hypertensive disorders of pregnancy; GDM = gestational diabetes mellitus; PTB = preterm birth; LBW = low birth weight; SGA = small for gestational age; LGA = large for gestational age.

*There were significant differences among groups.

^a^ There were significant differences between NC and HRT group.

^b^ There were significant differences between NC and OI group.

^c^ There were significant differences between HRT and OI group.

Supplementary table 10 Univariate and multivariate logistic regression model about NC, HRT and OI protocols in the IVF group

|  | Crude OR (95% Cl) | *P*-value | Adjusted OR (95% Cl) | *p*-value |
| --- | --- | --- | --- | --- |
| **HDP** |  |  |  |  |
| NC | 1 |  | 1 |  |
| HRT | 2.41 (1.81-3.21) | <0.001^*^ | 1.94 (1.41-2.67) | <0.001^*^ |
| OI | 1.39 (0.83-2.31) | 0.209 | 1.02 (0.58-1.79) | 0.959 |
| **GDM** |  |  |  |  |
| NC | 1 |  | 1 |  |
| HRT | 1.46 (1.10-1.93) | 0.008^*^ | 1.28 (0.93-1.77) | 0.133 |
| OI | 1.63 (1.08-2.46) | 0.020^*^ | 1.57 (0.99-2.49) | 0.054 |
| **Placenta previa** |  |  |  |  |
| NC | 1 |  | 1 |  |
| HRT | 1.26 (0.70-2.28) | 0.443 | 1.23 (0.64-2.38) | 0.540 |
| OI | 1.17 (0.46-3.00) | 0.740 | 1.30 (0.49-3.47) | 0.602 |
| **Oligohydramnios** |  |  |  |  |
| NC | 1 |  | 1 |  |
| HRT | 0.91 (0.46-1.80) | 0.792 | 0.81 (0.40-1.67) | 0.572 |
| OI | 2.00 (0.92-4.35) | 0.080 | 1.47 (0.62-3.49) | 0.387 |
| **PTB** |  |  |  |  |
| NC | 1 |  | 1 |  |
| HRT | 1.78 (1.36-2.34) | <0.001^*^ | 1.67 (1.24-2.27) | 0.001^*^ |
| OI | 1.55 (1.01-2.39) | 0.046^*^ | 1.30 (0.80-2.10） | 0.285 |
| **LBW** |  |  |  |  |
| NC | 1 |  | 1 |  |
| HRT | 1.47 (1.02-2.12) | 0.038^*^ | 1.23 (0.82-1.84) | 0.309 |
| OI | 0.86 (0.43-1.73) | 0.676 | 0.69 (0.33-1.42) | 0.313 |
| **SGA** |  |  |  |  |
| NC | 1 |  | 1 |  |
| HRT | 0.79 (0.51-1.21) | 0.276 | 0.78 (0.49-1.24) | 0.293 |
| OI | 1.42 (0.83-2.43) | 0.205 | 1.36 (0.77-2.43) | 0.292 |
| **LGA** |  |  |  |  |
| NC | 1 |  | 1 |  |
| HRT | 1.15 (0.98-1.34) | 0.094 | 1.08 (0.90-1.29) | 0.428 |
| OI | 0.86 (0.66-1.12) | 0.248 | 0.86 (0.64-1.15) | 0.314 |

*Note:* CI = confidence interval; HDP = hypertensive disorders of pregnancy; GDM = gestational diabetes mellitus; PTB = preterm birth; LBW = low birth weight; SGA = small for gestational age; LGA = large for gestational age.

*There were significant differences among groups.

Adjustment included age, body mass index, irregular menstruation, FET cycle number, preconceptional fasting glucose, systolic pressure, diastolic pressure, endometrial thickness, testosterone level, anti-Müllerian hormone.

Supplementary table 11 The baseline characteristics in ICSI group

|  | NC | HRT | OI | *p*-value |
| --- | --- | --- | --- | --- |
|  | N=1653 | N=584 | N=162 |  |
| Maternal age (year) | 30.5±4.1 | 30.1±4.1 | 30.6±4.5 | 0.143 |
| BMI (kg/m^2^) | 22.5±3.3^a^ | 23.1±3.6 | 22.8±3.3 | <0.001^*^ |
| Indications for IVF, n (%) |  |  |  |  |
| Tubal factor | 611 (37.0) | 193 (33.0) | 44 (27.2) | 0.018^*^ |
| Male factor | 893 (54.0) | 309 (52.9) | 96 (59.3) | 0.355 |
| Combined factors | 64 (3.9) | 46 (7.9) | 13 (8.0) | <0.001^*^ |
| Others | 85 (5.1) | 36 (6.2) | 9 (5.6) | 0.607 |
| Irregular menstruation, n (%) | 92 (5.6)^a,b^ | 125 (21.4) | 41 (25.3） | <0.001^*^ |
| Donor sperm using, n (%) | 24 (1.5)^b^ | 10 (1.7)^c^ | 7 (4.3) | 0.027^*^ |
| FET cycle number | 1.2±0.5^a,b^ | 1.3±0.6^c^ | 1.5±0.7 | <0.001^*^ |
| No.of transferred embryos, n (%) |  |  |  |  |
| =1 | 1476 (89.3) | 528 (90.4) | 145(89.5) | 0.748 |
| ≥2 | 177 (10.7) | 56 (9.6) | 17 (10.5) | 0.748 |
| Vanishing twin gestation, n (%) | 39 (2.4) | 20 (3.4) | 4 (2.5) | 0.381 |
| PFG (mmol/L) | 5.2±0.5^b^ | 5.2±0.4 | 5.2±0.5 | 0.604 |
| Systolic pressure (mmHg) | 120.5±11.8^a,b^ | 120.6±11.4 | 121.5±11.3 | 0.538 |
| Diastolic pressure (mmHg) | 72.2±8.6^b^ | 72.6±8.4^c^ | 74.3±8.4 | 0.013^*^ |
| Endometrial thickness (mm) | 10.0±1.6^a,b^ | 9.6±1.5 | 9.4±1.7 | <0.001^*^ |
| AFC | 15.3±5.8^a^ | 16.3±6.7 | 15.9±6.3 | 0.002^*^ |
| Testosterone level (ng/dL) | 24.4±11.6^a^ | 26.1±11.9 | 25.3±13.3 | 0.009^*^ |
| AMH (ng/mL) | 4.7±4.3^a^ | 5.3±3.5 | 5.0±3.7 | 0.022* |

*Note:* NC = natural cycle; BMI = body mass index; FET = frozen embryo transfer; PFG = preconceptional fasting glucose; AFC = Antral follicle count; AMH = anti-müllerian hormone.

*There were significant differences among groups.

^a^ There were significant differences between NC and HRT group.

^b^ There were significant differences between NC and OI group.

^c^ There were significant differences between HRT and OI group.

Supplementary table 12 The comparison of outcomes in ICSI group

|  | NC | HRT | OI | *p*-value |
| --- | --- | --- | --- | --- |
|  | N=1653 | N=584 | N=162 |  |
| HDP, n (%) | 52(3.1)^a^ | 40 (6.8)^c^ | 6 (3.7) | 0.001^*^ |
| GDM, n (%) | 88 (5.3) | 28 (4.8) | 10 (6.2) | 0.764 |
| Placenta previa, n (%) | 10 (0.6) | 8 (1.4) | 1 (0.6) | 0.194 |
| Oligohydramnios, n (%) | 26 (1.6) | 6 (1.0) | 1 (0.6) | 0.431 |
| PTB, n (%) | 66 (4.0)^a,b^ | 44(7.5) | 14 (8.6) | <0.001^*^ |
| LBW, n (%) | 40 (2.4)^a,b^ | 29 (5.0) | 10 (6.2) | 0.001^*^ |
| SGA, n (%) | 66 (4.0) | 21 (3.6) | 10 (6.2) | 0.332 |
| LGA, n (%) | 361 (21.8) | 147 (25.2) | 38 (23.5) | 0.250 |

*Note:* HDP = hypertensive disorders of pregnancy; GDM = gestational diabetes mellitus; PTB = preterm birth; LBW = low birth weight; SGA = small for gestational age; LGA = large for gestational age.

*There were significant differences among groups.

^a^ There were significant differences between NC and HRT group.

^b^ There were significant differences between NC and OI group.

^c^ There were significant differences between HRT and OI group.

Supplementary table 13 Univariate and multivariate logistic regression model about NC, HRT and OI protocols in the ICSI group

|  | Crude OR (95% Cl) | *p*-value | Adjusted OR (95% Cl) | *p*-value |
| --- | --- | --- | --- | --- |
| **HDP** |  |  |  |  |
| NC | 1 |  | 1 |  |
| HRT | 2.26 (1.48-3.46) | <0.001^*^ | 2.08 (1.30-3.33) | 0.002^*^ |
| OI | 1.18 (0.50-2.80) | 0.700 | 0.89 (0.34-2.34) | 0.810 |
| **GDM** |  |  |  |  |
| NC | 1 |  | 1 |  |
| HRT | 0.90 (0.58-1.39) | 0.620 | 0.81 (0.50-1.33) | 0.407 |
| OI | 1.17 (0.60-2.30) | 0.648 | 1.27 (0.62-2.59) | 0.515 |
| **Placenta previa** |  |  |  |  |
| NC | 1 |  | 1 |  |
| HRT | 2.28 (0.90-5.81) | 0.084 | 2.71 (0.97-1.58) | 0.057 |
| OI | 1.02 (0.13-8.02) | 0.985 | 1.32 (0.15-11.44) | 0.799 |
| **Oligohydramnios** |  |  |  |  |
| NC | 1 |  | 1 |  |
| HRT | 0.65 (0.27-1.59) | 0.344 | 0.57 (0.22-1.45) | 0.237 |
| OI | 0.39 (0.05-2.88) | 0.355 | 0.38 (0.05-2.95) | 0.357 |
| **PTB** |  |  |  |  |
| NC | 1 |  | 1 |  |
| HRT | 1.96 (1.32-2.91) | 0.001^*^ | 2.00 (1.30-3.08) | 0.002^*^ |
| OI | 2.28 (1.25-4.15) | 0.007^*^ | 1.98 (1.00-3.92） | 0.050 |
| **LBW** |  |  |  |  |
| NC | 1 |  | 1 |  |
| HRT | 2.11 (1.29-3.43) | 0.003^*^ | 2.18 (1.28-3.72) | 0.004^*^ |
| OI | 2.65 (1.30-5.41) | 0.007^*^ | 2.79 (1.29-6.03) | 0.009^*^ |
| **SGA** |  |  |  |  |
| NC | 1 |  | 1 |  |
| HRT | 0.90 (0.54-1.48) | 0.670 | 0.96 (0.56-1.65) | 0.891 |
| OI | 1.58 (0.80-3.14) | 0.190 | 1.93 (0.93-4.00) | 0.077 |
| **LGA** |  |  |  |  |
| NC | 1 |  | 1 |  |
| HRT | 1.20 (0.97-1.50) | 0.099 | 1.13 (0.94-1.68) | 0.056 |
| OI | 1.10 (0.75-1.61) | 0.635 | 1.26 (0.82-1.93) | 0.287 |

*Note:* CI = confidence interval; HDP = hypertensive disorders of pregnancy; GDM = gestational diabetes mellitus; PTB = preterm birth; LBW = low birth weight; SGA = small for gestational age; LGA = large for gestational age.

*There were significant differences among groups.

Adjustment included age, body mass index, tubal factor, combined factors, irregular menstruation, donor sperm using, FET cycle number, diastolic pressure, endometrial thickness, antral follicle count, testosterone level, anti-Müllerian hormone.
